# Amorphous mesoporous GeO*_x_* anode for Na-ion batteries with high capacity and long lifespan

**DOI:** 10.1098/rsos.171477

**Published:** 2018-01-17

**Authors:** Kangze Shen, Ning Lin, Tianjun Xu, Ying Han, Yitai Qian

**Affiliations:** Department of Chemistry, University of Science and Technology of China, Hefei, Anhui Province, 230026, People's Republic of China

**Keywords:** amorphous, mesoporous, GeO*_x_*, Na-ion batteries, high capacity, long lifespan

## Abstract

It is recently demonstrated that amorphous Ge anode shows higher reversible Na-ion storage capacity (590 mA h g^−1^) than crystallized Ge anode (369 mA h g^−1^). Here, amorphous GeO*_x_* anode is prepared by a simple wet-chemistry reduction route at room temperature. The obtained amorphous GeO*_x_* shows a porous hierarchical architecture, accompanied with a Brunauer–Emmett–Teller surface area of 159 m^2^ g^−1^ and an average pore diameter of 14 nm. This unique structure enables the GeO*_x_* anode to enhance the Na-ion/electron diffusion rate, and buffer the volume change. As anode for Na-ion battery, high reversible capacity over 400 mA h g^−1^, fine rate capability with a capacity of 200 mA h g^−1^ maintained at 3.0 A g^−1^ and long-term cycling stability with 270 mA h g^−1^ even over 1000 cycles at 1.0 A g^−1^ are obtained.

## 1. Introduction

Rechargeable sodium-ion batteries (NIBs) are regarded as intriguing energy storage systems, which have attracted increasing attention due to the natural abundance of metal sodium [1–3]. To date, the main research interests concerning NIBs are focused on fabricating various cathode/anode electrode materials which play a decisive role in the electrochemical performance such as specific capacity and cycling stability [4–8]. However, most electrode materials that have been developed for Li-ion batteries (LIBs) are generally unable to work for NIBs, because the size of Na^+^ ion (1.02 Å in radius) is larger than that of Li^+^ ion (0.59 Å in radius) [9,10]. With regard to the anode materials, the graphite anode in LIBs shows poor Na-ion storage performance owing to the graphitic lattice mismatching [11]. It is, therefore, highly desirable to explore suitable anode materials for facilitating the development of NIBs [12,13].

In analogy with LIBs, various alloy-type materials such as Si, Ge, Sn, Pb and Sb have been proposed as anodes for NIBs [14–21]. These anode materials can alloy with sodium at room temperature, exhibiting high reversible capacity. As for Ge anode materials, Chevrier & Ceder [1] calculated that germanium (Ge) could alloy with Na at room temperature to form NaGe, and the corresponding theoretical capacity is 369 mA h g^−1^. Then, several experimental results were reported [22,23]. For example, thin Ge film deposition on roughened Cu foils, prepared by DC magnetron sputtering, exhibited a reversible capacity of 350 mAh g^−1^ at C/20 (approx. 20 mA g^−1^), accompanied with Na alloy/dealloy reaction potentials at around 0.15/0.6 V [22].

Recently, Kohandehghan *et al.* [24] demonstrated that amorphization of Ge thin film anode by a prior lithiation–delithiation cycle would lead to a dramatic improvement for Na-ion storage performance such as high specific capacity, good rate capability and long-life cycling stability. It is reported that amorphization of Ge anode is able to lower the barrier for nucleation of the Na*_x_*Ge phase(s) and accelerate solid-state ionic diffusion. Accordingly, the activated amorphous Ge thin film delivered a reversible capacity of 418 mA h g^−1^ at 0.15C (1C = 369 mA g^−1^) after 50 cycles. Subsequently, Korgel *et al.* reported that amorphous Ge nanowires can provide a reversible capacity as high as 590 mAh g^−1^ by forming a sodiated phase of Na_1.6_Ge rather than NaGe [25]. The related measurements further suggested that the high defect density of amorphous Ge enables more Na ion to alloy with Ge at room temperature. Meanwhile, *in situ* transmission electron microscopy (TEM) imaging indicated that germanium nanowires exhibit a 300% expansion in volume upon sodiation, which is bad for the electrochemical performance.

Generally, fabricating oxide anodes such as SiO, SnO_*x*_ and GeO*_x_* is an effective route to address the volume change issues [26–33]. Typically, amorphous GeO*_x_* was used as anode for high-performance LIBs, and, recently, explored for NIBs. Kajita *et al.* [30] prepared amorphous GeO*_x_* (*x* < 1) powder by oxidizing Zintl-phase NaGe with isopropyl alcohol at room temperature, showing an initial reversible capacity 342 mAh g^−1^ at 0.2 A g^−1^ and with a capacity retention of 216 mAh g^−1^ after 40 cycles. The generated Na_2_O can act as a buffering phase to accommodate the volume change during cycling [31,32]. Although GeO*_x_* shows great potential performance for Na-ion storage, related reports are limited.

In this work, amorphous GeO*_x_* anode for NIBs was synthetized by a modified wet-chemistry reduction route [34–36] at room temperature that is simple and can be easily scaled up. The obtained amorphous GeO*_x_* shows a hierarchical mesoporous structure. The Brunauer–Emmett–Teller (BET) surface area is about 159 m^2^ g^−1^, the average pore diameter is about 14.0 nm ranging from 5 to 30 nm, with a pore volume of 0.352 m^3^ g^−1^. When the as-prepared GeO*_x_* material was used as an anode for rechargeable NIBs, high reversible capacity over 400 mAh g^−1^, fine rate capability of 200 mAh g^−1^ maintained even at 3.0 A g^−1^ and long-term cycling stability with 270 mAh g^−1^ after 1000 cycles at 1.0 A g^−1^ were obtained. The good Na-ion storage performance may be attributed to the unique amorphous hierarchical porous structure that could facilitate Na ion and electron diffusion, and buffer the volume change.

## 2. Experimental

**Figure 1.**
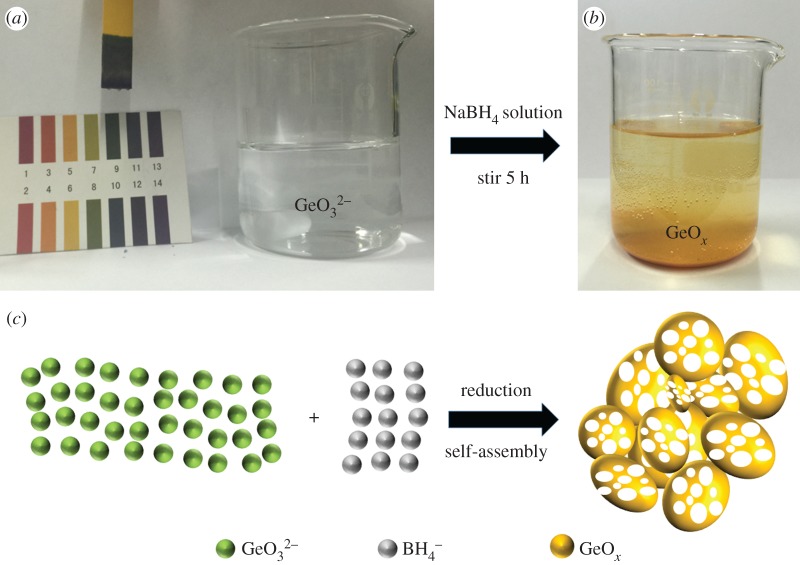
(*a*) The transparent solution after NaOH is added and the present main ingredient ${\text{GeO}}_{3}{}^{2-}$ ions are formed from GeO_2_, (*b*) the obtained yellow powder, GeO*_x_*, was collected after washing and drying, (*c*) the sketch of the main process.

The synthesis of amorphous GeO*_x_* sample was carried out by a wet-chemistry reduction route at room temperature, modifying a procedure reported by Han *et al.* [26]. In a typical experiment, 1.0 g of GeO_2_ was stirred in 100 ml of distilled water and dissolved by NaOH solution (0.1 M) added drop by drop under vigorous magnetic stirring at room temperature. After the dispersion became transparent, which is shown in figure 1*a*, fresh NaBH_4_ (98%, Alfa) solution (2 g in 20 ml of distilled water) was injected into the transparent solution, and the mixture was stirred continuously for 5 h. Finally, the obtained yellow powder which is presented in figure 1*b* was collected by filtration, washed with distilled water, and dried at 40°C under vacuum. Figure 1*c* shows a sketch of the main process. As a contrast experiment, crystallized GeO_2_/Ge composite was obtained by annealing the as-synthesized amorphous GeO*_x_* at 700°C under Ar atmosphere. The details of materials characterization and electrochemical measurements are provided in the electronic supplementary material.

## 3. Results and discussion

**Figure 2.**
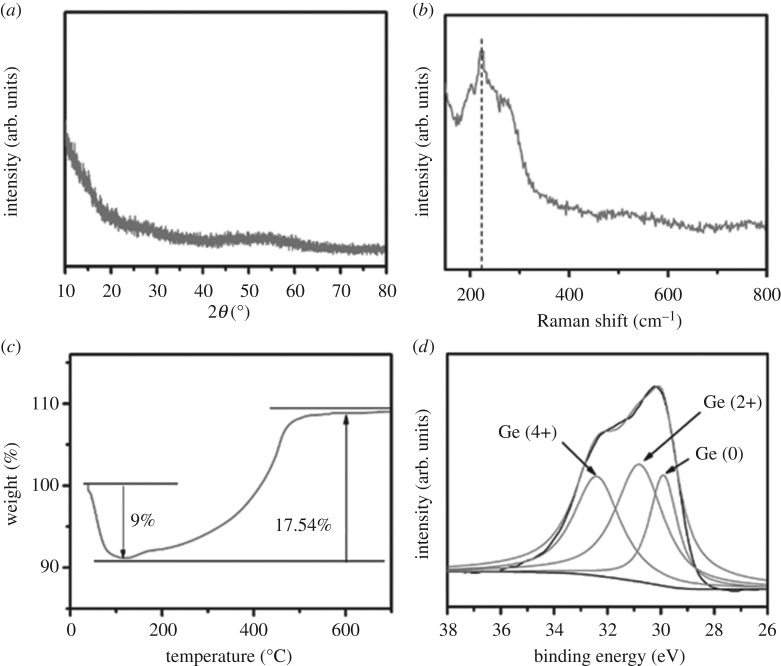
(*a*) XRD pattern, (*b*) Raman shift, (*c*) TGA curve in air atmosphere and (*d*) XPS Ge (3d) spectra of the as-prepared GeO*_x_* sample.

Figure 2*a* shows the X-ray diffraction (XRD) pattern of the obtained final product. Clearly, no diffraction peak can be observed, indicating amorphous structure of the synthesized sample. After annealing this sample at 700°C under Ar atmosphere, well crystallized Ge and GeO_2_ composites can be detected, as evident from the XRD patterns exhibited in electronic supplementary material, figure S2. Figure 2*b* shows the Raman spectrum of the amorphous sample. The band at around 240 cm^−1^ is ascribed to the typical Raman mode of amorphous Ge.

Figure 2*c* shows the thermal gravimetric analysis (TGA) plot. After heat treatment over 700°C under air atmosphere, all the Ge element including Ge (0) and Ge (2+) would be oxidized into Ge (4+), that is, the GeO*_x_* was converted into GeO_2_ completely. Based on the weight change in the TGA plot, the value of ‘*x*’ is calculated to be about 0.6. The X-ray photoelectron spectroscopy (XPS) Ge 3d spectrum of the amorphous sample shows several oxidation states (figure 2*d*). The three peaks at 32.8, 31.4 and 29.8 eV are assigned as Ge (+4), Ge (+2) and Ge (0), respectively [30]. The weight ratio of Ge and O atoms is determined to be 1/0.6 on basis of the XPS result, which is consistent with the TGA result.

**Figure 3.**
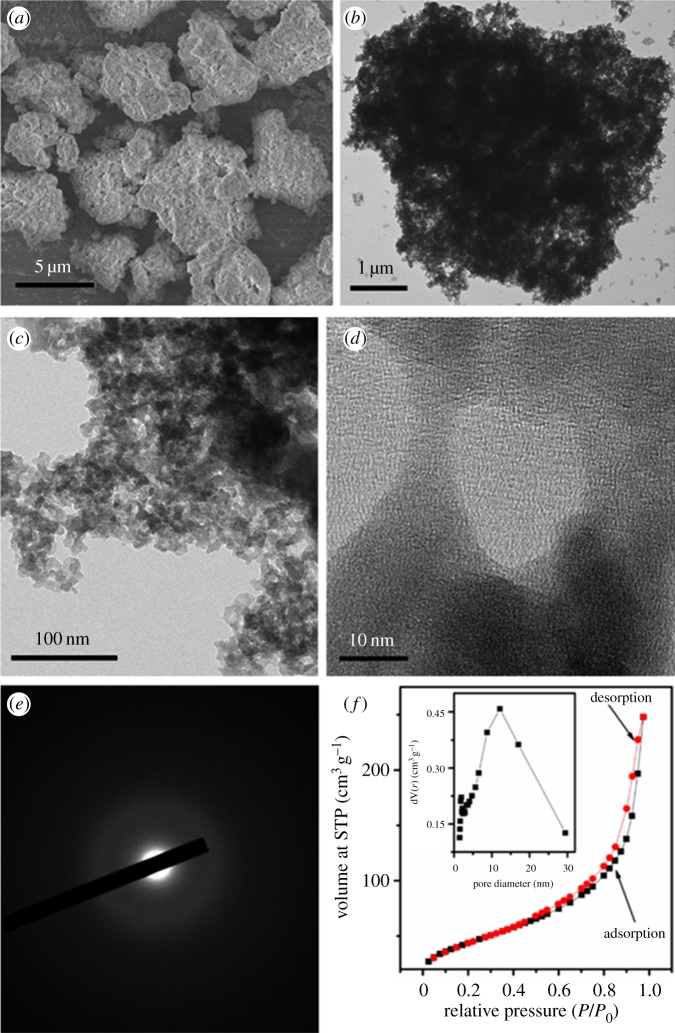
(*a*) SEM image, (*b*) low-magnification TEM image, (*c*) enlarged TEM image, (*d*) high-resolution TEM image, (*e*) SAED pattern and (*f*) nitrogen adsorption–desorption isotherms and BJH pore-size distribution of as-prepared GeO*_x_* sample.

Figure 3*a–d* shows the scanning electron microscopy (SEM) and TEM images of the amorphous GeO*_x_*. As one can see, the as-prepared product consists of micrometre-sized agglomerates composed of interconnected nanoparticles. The enlarged TEM image indicates that mesoscale pores are formed throughout this hierarchical structure. In the high-resolution TEM image which is shown in figure 3*d*, only amorphous phase matter can be observed. The selected area electron diffraction (SAED) pattern presented in figure 3*e* also proves an amorphous phase.

Figure 3*f* shows the nitrogen adsorption and desorption isotherms at 77 K, accompanied with the pore diameter distribution plot. The calculated BET specific surface area of GeO*_x_* is 159 m^2^ g^−1^. A Barrett–Joyner–Halenda (BJH) analysis of the nitrogen desorption curve indicated that the pore-size distribution of the GeO*_x_* ranges from 5 to 30 nm with an average pore size of 14.0 nm; the pore volume is calculated to be about 0.352 m^3^ g^−1^ (figure 3*f* inset). The above parameters all show that the obtained product grown from a wet-chemistry reduction route is a kind of mesoporous material. During the wet-chemistry process, the ${\text{GeO}}_{3}{}^{2-}$ is well dissolved in the solution, and when NaBH_4_ solution is injected, the ${\text{GeO}}_{3}{}^{2-}$ ions are reduced into little particles. Then, these irregular particles spontaneously aggregate and agglomerate, forming a large amount of void space. Besides, generated H_2_ bubbles are dispersed on the surface of nanoparticles, which is also facilitating the formation of porous structure.

**Figure 4.**
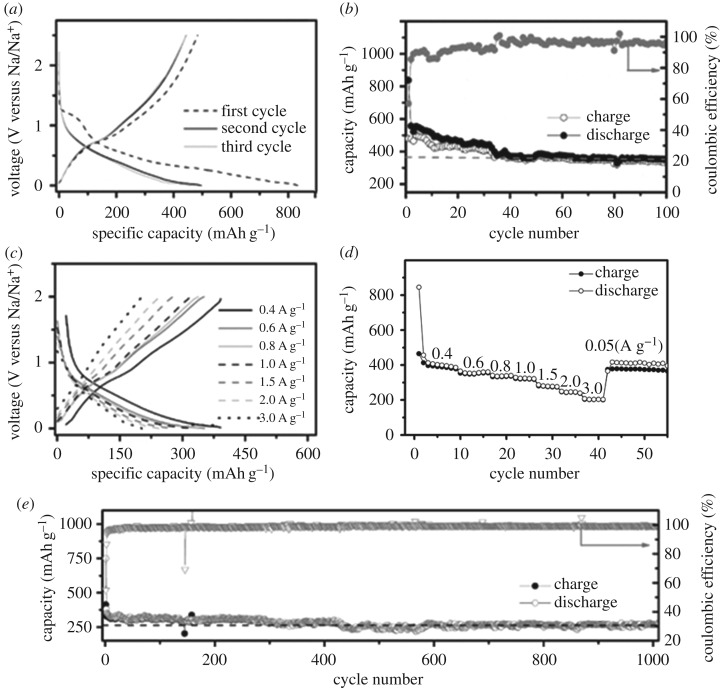
The Na-ion storage performance of the GeO*_x_* electrode. (*a*) The discharge/charge voltage profiles, and (*b*) cycling performance at 0.05 A g^−1^. (*c*) The discharge/charge voltage profiles, and (*d*) cycling performance at current density ranging from 0.05 to 3.0 A g^−1^. (*e*) The long-term cycling properties at 1.0 A g^−1^.

Figure 4*a* exhibits the galvanostatic discharge/charge voltage profiles at a current density of 0.05 A g^−1^. In the first discharge process, a distinct voltage plateau at 1.2 V (versus Na/Na^+^) is corresponding to the serious decomposition of electrolyte. As the Na-alloying reaction proceeds, a smooth discharging plot is observed. In the charging process, a voltage plateau at around 0.7 V (versus Na/Na^+^) agrees well with the cyclic voltammetry result (electronic supplementary material, figure S1 inset). The initial discharge and charge capacities of the GeO*_x_* electrode are 834 and 476 mAh g^−1^, respectively. The corresponding initial coulombic efficiency is as low as 57%, that is, a large amount of Na^+^ is irreversibly consumed. The large capacity loss is generally caused by the irreversible formation of solid--electrolyte interphase (SEI) film/Na_2_O, and the Na^+^ alloying reaction. From second cycle forwards, the charge/discharge voltage plateau is replaced by a group of sloping plots. Noteworthy, the irreversible capacity decreased remarkably, suggesting a stable SEI film was generated.

Figure 4*b* shows the cycling performance of the GeO*_x_* electrode at a current density of 0.05 A g^−1^. The first reversible capacity is 470 mAh g^−1^. Owing to the large specific surface area, the GeO*_x_* electrode needs tens of cycles to form a good SEI layer. After 100 cycles, the GeO*_x_* electrode maintains a specific capacity of 350 mAh g^−1^ that is 74% of the first capacity. As a contrast, the crystallized Ge/GeO_2_ electrode exhibits low Na-ion storage performance, delivering an initial reversible capacity of 65 mAh g^−1^ at 0.05A g^−1^, accompanied with an initial coulombic efficiency of 24% (electronic supplementary material, figure S4 inset). The Na-storage capacity of Super-P carbon black of about 120 mAh g^−1^ is noted (electronic supplementary material, figure S6 inset). It is reasonable to conclude that the amorphous phase Ge could enhance the Na-alloying reaction during discharge/charge cycling.

The rate capability of the as-prepared amorphous GeO*_x_* electrode is evaluated with the current density ranging from 0.05 to 3.0 A g^−1^. Figure 4*c,d* exhibits the galvanostatic charge/discharge voltage profiles for the last cycle and cycling properties at different current densities, respectively. The GeO*_x_* delivered a reversible capacity of 382, 350, 333, 324, 278, 245 and 202 mAh g^−1^ at a current density of 0.4, 0.6, 0.8, 1.0, 1.5, 2.0 and 3.0 A g^−1^, respectively. As one can see, the GeO*_x_* electrode still delivers a reversible capacity of 200 mAh g^−1^ even at high current density of 3.0 A g^−1^. After high-rate cycling, a reversible capacity of 380 mAh g^−1^ is recovered as the current density returned back to 0.05 A g^−1^. It should be pointed out that the overpotential during discharge/charge becomes more and more apparent as the current densities increased.

Figure 4*e* displays the long-term cycling stability at a current density of 1.0 A g^−1^. Note that the initial two cycles of the coin cell are tested at a low current density of 0.2 A g^−1^ in order to activate the electrode sufficiently. At the current density of 1.0 A g^−1^, the GeO*_x_* shows a specific capacity of 300 mAh g^−1^. From 100th cycle forward, no remarkable capacity fading is observed. After 1000 cycles, the obtained reversible capacity is maintained as high as 270 mAh g^−1^, indicating fine long-term cycling stability.

**Figure 5.**
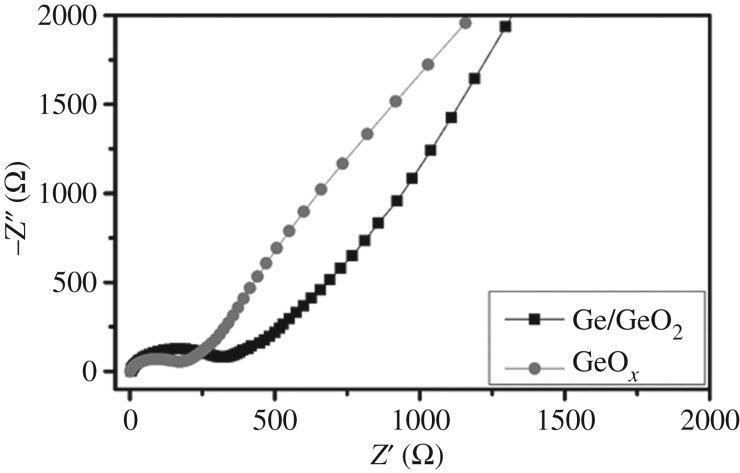
The Nyquist plots of amorphous GeO*_x_* and GeO_2_/Ge electrodes.

Electrochemical impedance spectroscopy measurements were conducted to further investigate the electrochemical performance. Figure 5 shows the experimental Nyquist plots of the amorphous GeO*_x_* and GeO_2_/Ge electrodes before cycling. All of the Nyquist plots consist of a semicircle in the high frequency region, and a straight line in the low frequency region. The diameter of the semicircle is ascribed to the charge transfer resistance on the interface of the electrolyte and active material, while the linear region corresponds to the lithium ion diffusion in the electrodes. As one can see, the amorphous GeO*_x_* electrode exhibits lower charge transfer impedance than the crystalline Ge/GeO_2_ electrode, because the highly porous surface structure of the amorphous GeO*_x_* facilitates ion/electron diffusion.

Above all, the superior electrochemical performance such as high reversible capacity, fine rate capability and long-term cycling life of the as-synthesized GeO*_x_* is closely related to synergistic effects of the amorphous hierarchical structure. First, the amorphous phase makes the electrochemical sodiation/desodiation of GeO*_x_* electrode to occur easily, which could store more Na^+^ than that of crystallized Ge anode, providing high specific capacity [25]. Second, the porous structure and high surface area can provide fast ionic/electronic transport path and enhance the electrochemical reactivity [29]. Third, the hierarchical porous architecture can not only afford high opening void space, but also combine the advantageous properties of microscale and mesoscale structure, which enables the anode to release the strain stress and accommodate the volume change. Therefore, the particle integrity and electrical contact of active materials in the electrode could be well preserved during repeated Na-alloy/dealloy reaction. What is more, the generated inactive Na_2_O during initial discharge process could also act as a buffering phase to address the volume expansion [32].

## 4. Conclusion

In summary, amorphous GeO*_x_* anode material for NIBs was fabricated by a modified room-temperature wet-chemical approach. The obtained amorphous GeO*_x_* exhibits a porous hierarchical structure composed of interconnected nanoparticles. The BET surface area and the average pore diameter were 159 m^2^ g^−1^ and 14 nm, respectively. The amorphous GeO*_x_* shows high electrochemical sodiation reactivity, and the porous hierarchical structure is beneficial for buffering the volume change and the ionic/electronic conductivity during discharge/charge process. As a result, a high reversible capacity over 400 mAh g^−1^, good rate capability with 200 mAh g^−1^ at 3.0 A g^−1^ and long-term cycling life over 1000 cycles are obtained. This work paves a novel route for exploring high-performance anode materials for rechargeable NIBs.

## Supplementary Material

All supplementary materials

## Supplementary Material

Figure S1

## Supplementary Material

Figure S2

## Supplementary Material

Figure S3

## Supplementary Material

Figure S4

## Supplementary Material

Figure S5

## Supplementary Material

Figure S6

## Data accessibility

We include all the experimental data in the electronic supplementary material.

## References

[1] Chevrier VL, Ceder G 2011 Challenges for Na-ion negative electrodes. J. Electrochem. Soc. 158, A1011–A1014. (doi:10.1149/1.3607983)

[2] Kundu D, Talaie E, Duffort V, Nazar LF 2015 The emerging chemistry of sodium ion batteries for electrochemical energy storage. Angew. Chem. Int. Ed. 54, 3431–3448. (doi:10.1002/anie.201410376) 10.1002/anie.201410376 25653194

[3] Yadegari H, Sun Q, Sun X 2016 Sodium-oxygen batteries: a comparative review from chemical and electrochemical fundamentals to future perspective. Adv. Mater. 28, 7065–7093. (doi:10.1002/adma.201504373) 27258965 10.1002/adma.201504373

[4] You Y et al. 2016 Subzero-temperature cathode for a sodium-ion battery. Adv. Mater. 28, 7243–7248. (doi:10.1002/adma.201600846) 27305570 10.1002/adma.201600846

[5] Balogun MS, Luo Y, Qiu W, Liu P, Tong Y 2016 A review of carbon materials and their composites with alloy metals for sodium ion battery anodes. Carbon 98, 162–178. (doi:10.1016/j.carbon.2015.09.091)

[6] Dugas R, Zhang B, Rozier P, Tarascon JM 2016 Optimization of Na-ion battery systems based on polyanionic or layered positive electrodes and carbon anodes. J. Electrochem. Soc. 163, A867–A874. (doi:10.1149/2.0051605jes)

[7] Wang S, Wang L, Zhu Z, Hu Z, Zhao Q, Chen J 2014 All organic sodium-ion batteries with Na_4_C_8_H_2_O_6_. Angew. Chem. 126, 6002–6006. (doi:10.1002/ange.201400032) 10.1002/anie.201400032 24677513

[8] Palomares V, Serras P, Villaluenga I, Hueso KB, Carretero-González J, Rojo T 2012 Na-ion batteries, recent advances and present challenges to become low cost energy storage systems. Energy Environ. Sci. 5, 5884–5901. (doi:10.1039/C2EE02781J)

[9] Wang LP, Yu L, Wang X, Srinivasan M, Xu ZJ 2015 Recent developments in electrode materials for sodium-ion batteries. J. Mater. Chem. A 3, 9353–9378. (doi:10.1039/C4TA06467D)

[10] Park Y-U, Seo D-H, Kwon H-S, Kim B, Kim J, Kim H, Kim I, Yoo H-I, Kang K 2013 A new high-energy cathode for a Na-ion battery with ultrahigh stability. J. Am. Chem. Soc. 135, 13 870–13 878. (doi:10.1021/ja406016j) 10.1021/ja406016j 23952799

[11] Nobuhara K, Nakayama H, Nose M, Nakanishi S, Iba H 2013 First-principles study of alkali metal-graphite intercalation compounds. J. Power Sources 243, 585–587. (doi:10.1016/j.jpowsour.2013.06.057)

[12] Mortazavi M, Ye Q, Birbilis N, Medhekar NV 2015 High capacity group-15 alloy anodes for Na-ion batteries: electrochemical and mechanical insights. J. Power Sources 285, 29–36. (doi:10.1016/j.jpowsour.2015.03.051)

[13] Liu Y, Zhang N, Jiao L, Tao Z, Chen J 2015 Ultrasmall Sn nanoparticles embedded in carbon as high-performance anode for sodium-ion batteries. Adv. Funct. Mater. 25, 214–220. (doi:10.1002/adfm.201402943)

[14] Kim H, Kim H, Ding Z, Lee MH, Lim K, Yoon G, Kang K 2016 Recent progress in electrode materials for sodium-ion batteries. Adv. Energy Mater. 6, 1600943 (doi:10.1002/aenm.201600943)

[15] Jung SC, Jung DS, Choi JW, Han Y-K 2014 Atom-level understanding of the sodiation process in silicon anode material. J. Phys. Chem. Lett. 5, 1283–1288. (doi:10.1021/jz5002743) 26274485 10.1021/jz5002743

[16] Darwiche A, Marino C, Sougrati MT, Fraisse B, Stievano L, Monconduit L 2012 Better cycling performances of bulk Sb in Na-ion batteries compared to Li-ion systems: an unexpected electrochemical mechanism. J. Am. Chem. Soc. 134, 20 805–20 811. (doi:10.1021/ja310347x) 10.1021/ja310347x 23194439

[17] Yue C et al. 2015 High performance 3D Si/Ge nanorods array anode buffered by TiN/Ti interlayer for sodium-ion batteries. Adv. Funct. Mater. 25, 1386–1392. (doi:10.1002/adfm.201403648)

[18] Xu Y, Zhu X, Zhou X, Liu X, Liu Y, Dai Z, Bao J 2014 Ge nanoparticles encapsulated in nitrogen-doped reduced graphene oxide as an advanced anode material for lithium-ion batteries. J. Phys. Chem. C 118, 28 502–28 508. (doi:10.1021/jp509783h)

[19] Liu X, Du Y, Hu L, Zhou X, Li Y, Dai Z, Bao J 2015 Understanding the effect of different polymeric surfactants on enhancing the silicon/reduced graphene oxide anode performance. J. Phys. Chem. C 119, 5848–5854. (doi:10.1021/jp512152f)

[20] Hu L, Zhu X, Du Y, Li Y, Zhou X, Bao J 2015 A chemically coupled antimony/multilayer graphene hybrid as a high-performance anode for sodium-ion batteries. Chem. Mater. 27, 8138–8145. (doi:10.1021/acs.chemmater.5b03920)

[21] Xu X, Dou Z-F, Gu E, Si L, Zhou X, Bao J 2017 Uniformly-distributed Sb nanoparticles in ionic liquid-derived nitrogen-enriched carbon for highly reversible sodium storage. J. Mater. Chem. A 5, 13 411–13 420. (doi:10.1039/C7TA03434B)

[22] Baggetto L, Keum JK, Browning JF, Veith GM 2013 Germanium as negative electrode material for sodium-ion batteries. Electrochem. Commun. 34, 41–44. (doi:10.1016/j.elecom.2013.05.025)

[23] Abel PR, Lin Y-M, de Souza T, Chou C-Y, Gupta A, Goodenough JB, Hwang GS, Heller A, Mullins CB 2013 Nanocolumnar germanium thin films as a high-rate sodium-ion battery anode material. J. Phys. Chem. C 117, 18 885–18 890. (doi:10.1021/jp407322k)

[24] Kohandehghan A, Cui K, Kupsta M, Ding J, Memarzadeh Lotfabad E, Kalisvaart WP, Mitlin D 2014 Activation with Li enables facile sodium storage in germanium. Nano Lett. 14, 5873–5882. (doi:10.1021/nl502812x) 25233131 10.1021/nl502812x

[25] Lu X, Adkins ER, He Y, Zhong L, Luo L, Mao SX, Wang C-M, Korgel BA 2016 Germanium as a sodium ion battery material: *in situ* TEM reveals fast sodiation kinetics with high capacity. Chem. Mater. 28, 1236–1242. (doi:10.1021/acs.chemmater.6b00200)

[26] Shimizu M, Usui H, Fujiwara K, Yamane K, Sakaguchi H 2015 Electrochemical behavior of SiO as an anode material for Na-ion battery. J. Alloys Compd. 640, 440–443. (doi:10.1016/j.jallcom.2015.03.171)

[27] Zhao J et al. 2016 Metallurgically lithiated SiOx anode with high capacity and ambient air compatibility. Proc. Natl Acad. Sci. USA 113, 7408–7413. (doi:10.1073/pnas.1603810113) 27313206 10.1073/pnas.1603810113 PMC4941422

[28] Su D, Ahn H-J, Wang G 2013 SnO_2_@graphene nanocomposites as anode materials for Na-ion batteries with superior electrochemical performance. Chem. Commun. 49, 3131–3133. (doi:10.1039/C3CC40448J) 10.1039/c3cc40448j 23478677

[29] Wang X-L, Han W-Q, Chen H, Bai J, Tyson TA, Yu X-Q, Wang X-J, Yang X-Q 2011 Amorphous hierarchical porous GeO*x* as high-capacity anodes for Li ion batteries with very long cycling life. J. Am. Chem. Soc. 133, 20 692–20 695. (doi:10.1021/ja208880f) 10.1021/ja208880f 22141466

[30] Kajita T, Itoh T 2016 Electrochemical sodium storage in amorphous GeO*x* powder. Electrochim. Acta 195, 192–198. (doi:10.1016/j.electacta.2016.02.117)

[31] Kajita T, Itoh T 2016 Electrochemical performance of highly amorphous GeOx powders synthesized in different alcohols for use in Na and Li-ion batteries. RSC Adv. 6, 102 109–102 115. (doi:10.1039/C6RA20794D)

[32] Ngo DT, Kalubarme RS, Le HTT, Park C-N, Park C-J 2015 Conducting additive-free amorphous GeO_2_/C composite as a high capacity and long-term stability anode for lithium ion batteries. Nanoscale 7, 2552–2560. (doi:10.1039/C4NR05541A) 25579776 10.1039/c4nr05541a

[33] Lv D, Gordin ML, Yi R, Xu T, Song J, Jiang YB, Choi D, Wang D 2014 GeOx/reduced graphene oxide composite as an anode for Li-ion batteries: enhanced capacity via reversible utilization of Li_2_O along with improved rate performance. Adv. Funct. Mater. 24, 1059–1066. (doi:10.1002/adfm.201301882)

[34] Jing C, Zang X, Bai W, Chu J, Liu A 2009 Aqueous germanate ion solution promoted synthesis of worm-like crystallized Ge nanostructures under ambient conditions. Nanotechnology 20, 505607 (doi:10.1088/0957-4484/20/50/505607) 19934484 10.1088/0957-4484/20/50/505607

[35] Wu J, Sun Y, Zou R, Song G, Chen Z, Wang C, Hu J 2011 One-step aqueous solution synthesis of Ge nanocrystals from GeO_2_ powders. CrystEngComm 13, 3674–3677. (doi:10.1039/C1CE05191A)

[36] Wang Y, Wang G 2013 Facile synthesis of Ge@ C core–shell nanocomposites for high-performance lithium storage in lithium-ion batteries. Chem. Asian J. 8, 3142–3146. (doi:10.1002/asia.201300858) 24006143 10.1002/asia.201300858

