# Supplementary material for: Amorphous mesoporous GeOx anode for Na-ion batteries with high capacity and long lifespan
Source: R Soc Open Sci. 2018 Jan 17;5(1):171477. doi: 10.1098/rsos.171477 (PMC5792927; doi:10.1098/rsos.171477)
Supplement: All supplementary materials [file rsos171477supp1.docx]

**Supporting information**

**Amorphous mesoporous GeOx anode for Na-ion batteries with high capacity and long life-span**

Kangze Shen, Ning Lin*, Tianjun Xu, Ying Han, Yitai Qian

*Department of chemistry, university of science and technology of china, Hefei Anhui province, P. R. china.*

**Experimental section**

Structural characterization methods

XRD was performed on a Philips X’Pert PRO SUPER X-ray diffractometer equipped with graphite monochromatized Cu Kα radiation. Raman spectra was excited by radiation of 514.5 nm from a Jobin-Yvon (France) LABR AM-HR confocal laser micro-Raman spectrometer. SEM was operated on a ﬁeld emission scanning electron micro analyzer (Zeiss Supra 40) at the acceleration voltage of 5 kV. TEM images were obtained on a Hitachi H7650 transmission electron microscope with Change-Coupled Device (CCD) imaging system on the acceleration voltage of 120 kV. TGA was performed on a Perkin Elmer Diamond Thermogravimetry/Differential Thermal Analysis (TG/DTA) thermal analyzer with a heating rate of 10 °C min^-1^. The XPS spectra were recorded on an ESCALAB-MK-II X-ray photoelectron spectrometer using Mg Kα radiation exciting source (1253.6 eV). N_2_ sorption analysis was conducted on an ASAP 2020 accelerated surface area and porosimetry instrument (Micromeritics), equipped with automated surface area, at 77 K using Barrett-Emmett-Teller (BET) calculations for the surface area. The pore size distribution plot was recorded from the adsorption branch of the isotherm based on the Barrett-Joyner-Halenda (BJH) model.

Electrochemical measurement

The as-prepared GeOx samples were mixed with Super-P carbon black (Timcal) and Sodium alginate (SA) binder in weight ratio of 70:15:15 in deionized water solvent to form a slurry. The slurry was pasted on copper foil and dried in a vacuum oven at 60 ° C for 5 h. The active material density of each electrode was determined to be about 1.0~1.2mg cm^-2^. The copper foil electrode and the metallic sodium disc were assembled into 2016-type coin cells in which the Na discs were utilized as counter and reference electrode. The electrolyte is 1 M NaClO_4_ solution in a 50:50 (v/v) mixture of ethylene carbonate (EC) and diethyl carbonate (DEC) as the electrolyte, the separator is Whatman GF/C membrane. The whole assemble process was carried out in an Ar-filled glove box. Galvanostatic measurements were conducted using a LAND-CT2001A instrument at room temperature with a fixed voltage range of 0.01-2.0 V (*vs.* Na/Na^+^). The current density and the specific capacity was calculated on basis of the mass weight of active GeOx in the electrode. Cyclic voltammetry (CV) was performed on electrochemistry workstation (CHI660E), with a scanning rate of 0.1 mV s^-1^ at room temperature. The Electrochemical impedance spectroscopy (EIS) was also measured with an electrochemical workstation (CHI660E) by applying an alternating current (AC) voltage of 5 mV in the frequency range from 100 kHz to 0.1 Hz.

**
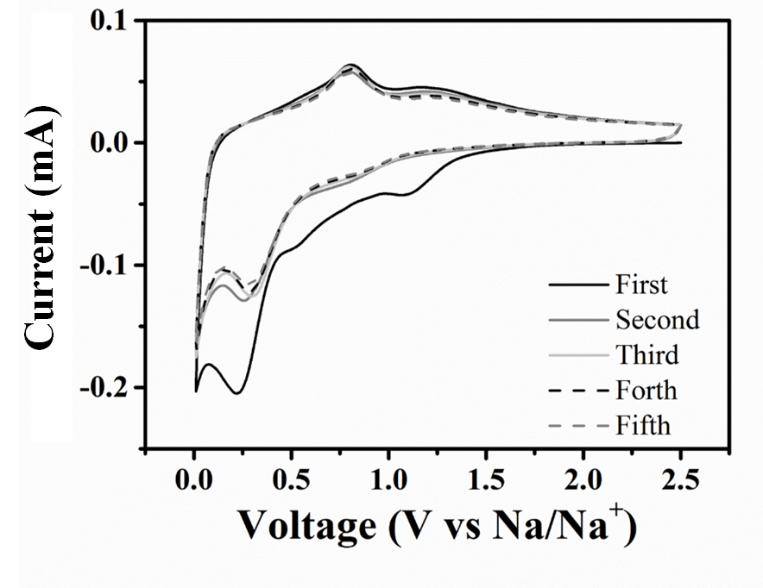
**

**Figure S1** The CV curves of the as-prepared GeOx electrode

The Na-ion storage performance of the as-synthesized amorphous GeOx is evaluated by a series of electrochemical measurements using coin-type half cells. Figure S1 exhibits the cyclic voltammogram (CV) with a scan rate of 0.1 mV s^-1^, in the voltage range of 0.01-2.5 V (vs Na/Na^+^). In the ﬁrst cycle, the reduction peak at 1.2 V and the broad peak at 0.5 V are attributed to forming solid electrolyte interface (SEI) membrane. And the two irreversible peaks at different potentials may rely on the decomposition of the electrolyte on different active surfaces of GeOx and Super-P carbon black. [1] We prove that the broad peak at 0.5 V is corresponding to the generation of solid electrolyte interface (SEI) membrane of Super-P carbon black by contrast trial (figure S5 inset) Both peaks are disappeared in the following cycles, implying irreversible reaction occurred in the initial cycles. The reaction peak below 0.2 V is assigned to alloying reaction between Ge and Na to produce Na_x_Ge. In the oxidation section, the peak at around 0.7 V is contributed to the de-alloying process of Na_x_Ge. The reduction-oxidation current peaks in subsequent CV curves are similar to first cycle and overlapped well, except for the disappearance of the initial reduction peak at 1.2 V. These results suggest fine electrochemically reversible Na-alloying/dealloying reaction of the amorphous GeOx electrode. Figure S3 shows the CV profiles of the crystallized GeO_2_/Ge electrode. Similarly, the initial reduction peaks at 1.2 V are observed. However, the Na alloying/dealloying reaction peaks are all located at around 0.1 V, which is different from the amorphous GeOx samples.


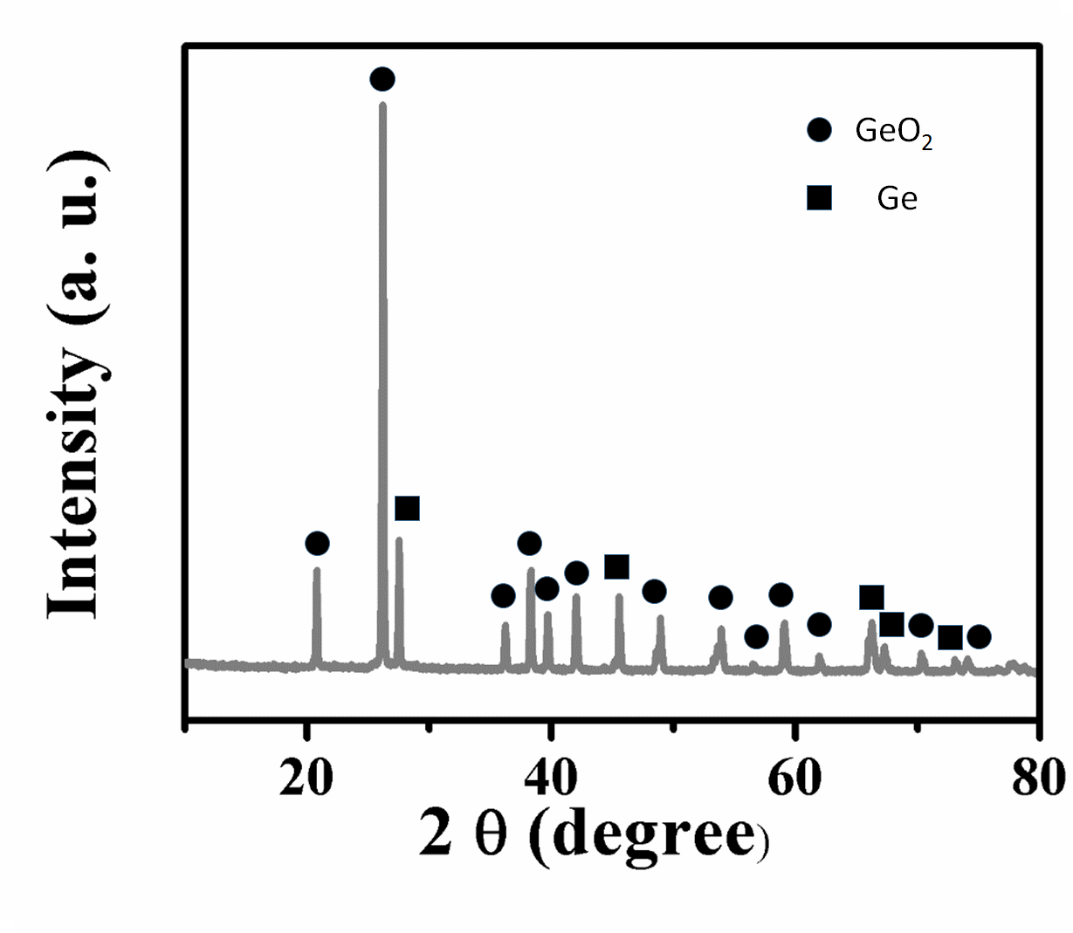


Figure S2 XRD pattern of the annealed GeOx sample.


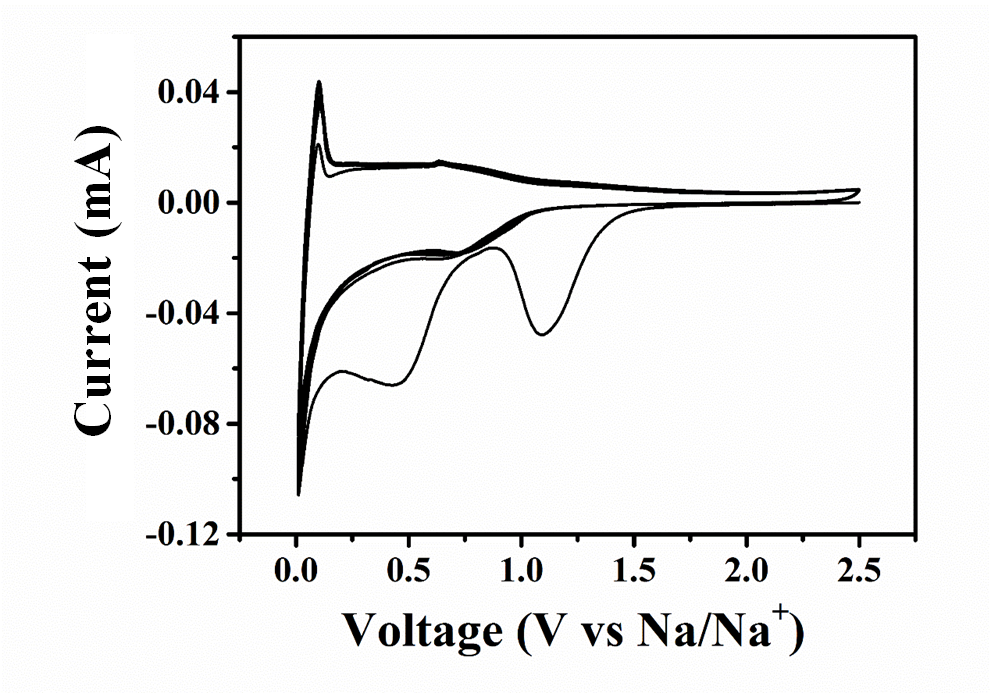


Figure S3 The CV curves of the as-prepared GeO_2_/Ge composite.





Figure S4 The cycling performance of the as-prepared crystallized GeO_2_/Ge composite at a current density of 0.05 A g^-1^.


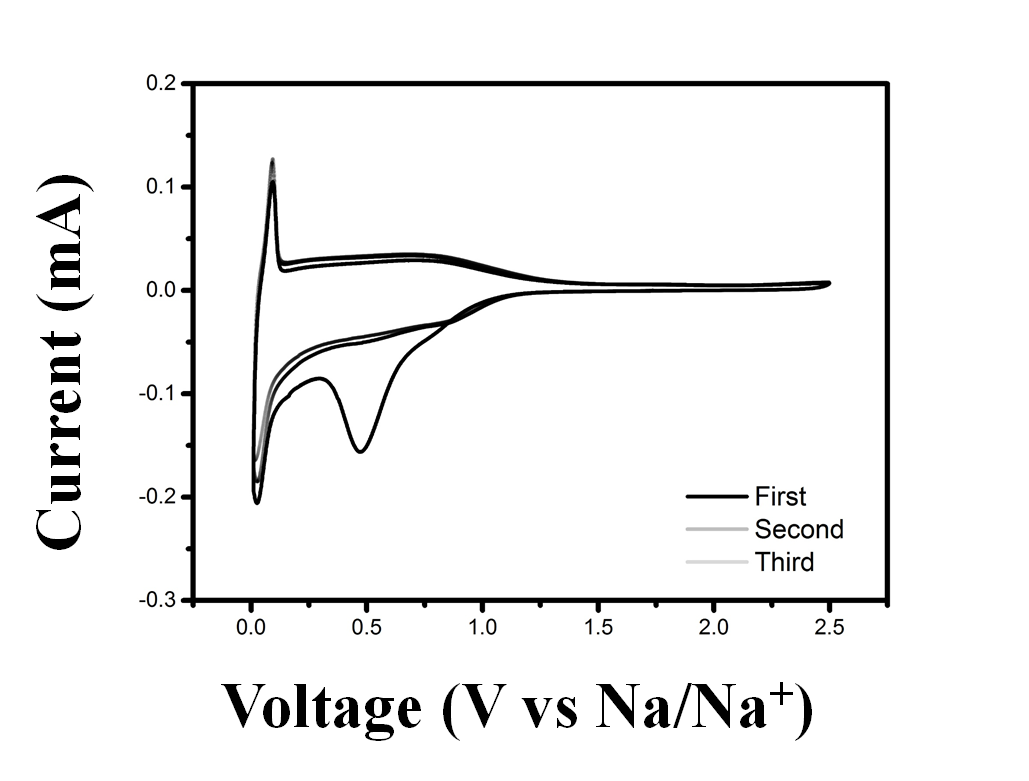


Figure S5 The CV curves of Super-P carbon black


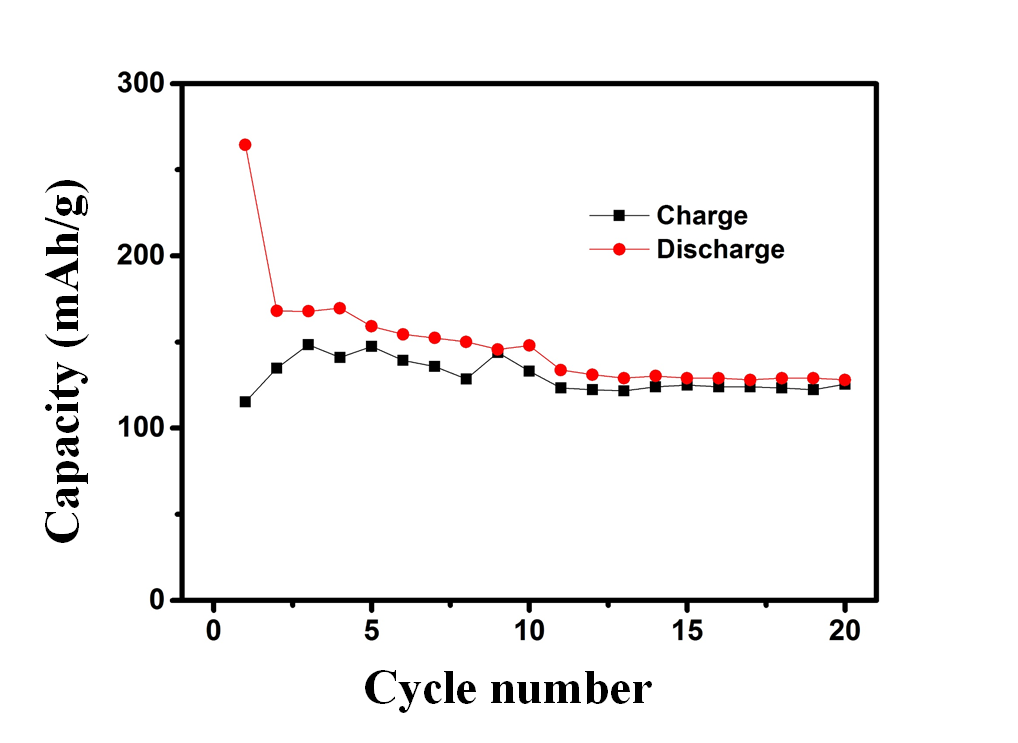


Figure S6 The cycling performance of the Super-P carbon black at a current density of 0.05 A g^-1^.

**References**

1. Cao, Y., Xiao, L., Sushko, M. L., Wang, W., Schwenzer, B., Xiao, J., Nie, Z., Saraf, L. V., Yang, Z., Liu, J. 2012 Sodium ion insertion in hollow carbon nanowires for battery applications. *Nano Lett.* **12**, 3783-3787. (doi:10.1021/nl3016957)
