# Supplementary figures and images for: Amorphous mesoporous GeOx anode for Na-ion batteries with high capacity and long lifespan
Source: R Soc Open Sci. 2018 Jan 17;5(1):171477. doi: 10.1098/rsos.171477 (PMC5792927; doi:10.1098/rsos.171477)

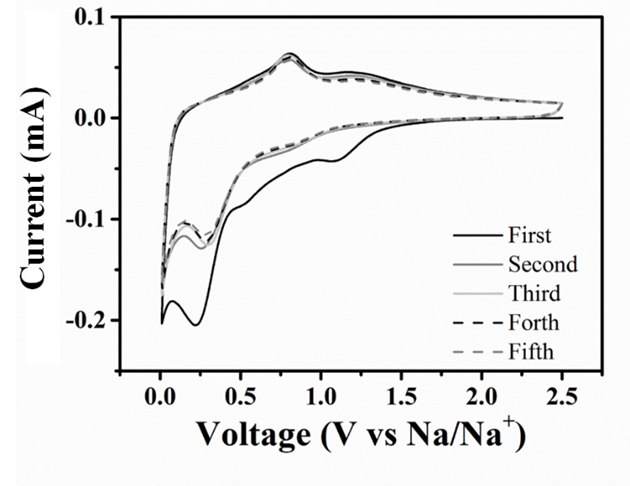

Supplement: Figure S1 [file rsos171477supp2.png]

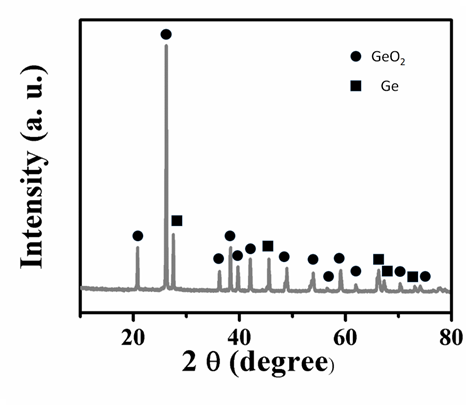

Supplement: Figure S2 [file rsos171477supp3.png]

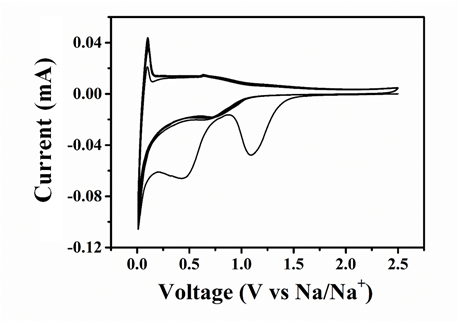

Supplement: Figure S3 [file rsos171477supp4.png]

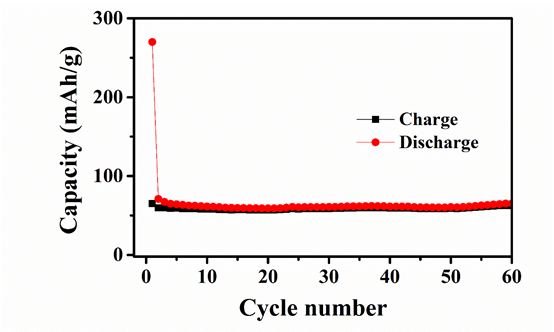

Supplement: Figure S4 [file rsos171477supp5.png]

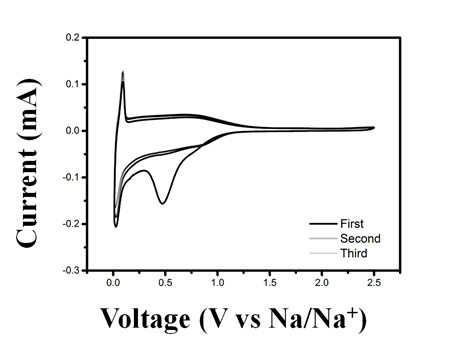

Supplement: Figure S5 [file rsos171477supp6.png]

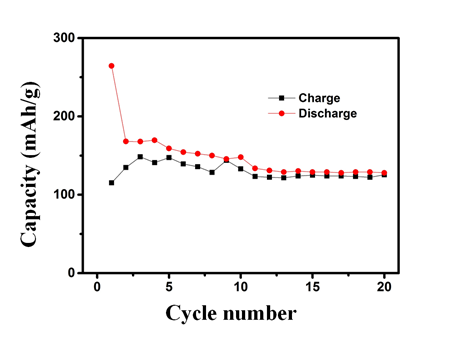

Supplement: Figure S6 [file rsos171477supp7.png]
